# Changes in Physical Fitness, Dietary Habits and Family Habits for Spanish Children during SARS-CoV-2 Lockdown

**DOI:** 10.3390/ijerph182413293

**Published:** 2021-12-16

**Authors:** Oliver Ramos Álvarez, Víctor Arufe Giráldez, David Cantarero Prieto, Alba Ibáñez García

**Affiliations:** 1 Education Faculty, Elviña University Campus, University of A Coruña, 15008 A Coruña, Spain; 2 Education Faculty, Physical Education and Sport Area, Interfacultative Building, University of Cantabria, 39005 Santander, Spain; 3 Research Unit of School Sports, Physical Education and Psychomotricity (UNIDEF), Specific Didactics Department, Research and Diagnostic Methods in Education, Education Faculty, Elviña University Campus, University of A Coruña, 15008 A Coruña, Spain; v.arufe@udc.es; 4 Health Economics and Health Services Management Research Group, IDIVAL Valdecilla, 39011 Santander, Spain; david.cantarero@unican.es (D.C.P.); alba.ibanez@unican.es (A.I.G.); 5 Department of Economics, University of Cantabria, 39006 Santander, Spain; 6 Education Faculty, Personality, Psychological Assessment and Treatments Area, Interfacultative Building, University of Cantabria, 39005 Santander, Spain

**Keywords:** SARS-CoV-2 lockdown, physical fitness, dietary habits, family habits, physical activity, Spanish children

## Abstract

Background: habits related to diet and physical activity in children were modified due to the lockdown that Spain had between March and June 2019 because of the health crisis caused by the appearance of SARS-CoV-2. The aim of the study was to know the impact that the lockdown had on physical fitness values in children aged 11–12. Methods: the study consisted of 50 Spanish children aged 11–12 (M = 11.40; SD = 0.50), 33 (66%) boys and 17 (34%) girls. Data collection was performed using the Alpha-Fitness Battery, a validated instrument to assess dietary intake, habits and practices, and an ad hoc survey to collect sociodemographic data and other information relevant to the study. Results: there were significant differences (*p* < 0.05) in the results of fitness variables measured in the standing long jump, agility speed and aerobic capacity, as well as in the results of maximal oxygen uptake (VO_2_ max) between, before and after lockdown in both boys and girls. No significant differences were found in measurements of right and left hand grip (*p* > 0.05). Conclusions: there is evidence of a significant impact of SARS-CoV-2 lockdown on physical fitness values in boys and girls aged 11–12.

## 1. Introduction

Spain was under a large-scale home lockdown between 15 March and 21 June 2020 due to the outbreak of the SARS-CoV-2 virus to prevent its spread throughout the country. The origin of this pandemic prompted the Spanish government as well as many other countries (e.g., Italy, France or Portugal) to establish various forms of home lockdown. In Spain, a total of 98 days of lockdown were decreed by Royal Decree, which limited the movement of all those who did not have essential jobs [1].

SARS-CoV-2 was first identified in Wuhan City (China) on 31 December 2019. This newly identified virus is a new type of coronavirus that infects and replicates in the pneumocytes and macrophages of the lung parenchyma where the ACE-2 cell receptor resides, causing symptoms of fever, dry cough, lymphopenia, dyspnoea and severe pneumonia that can lead to the death of the person who contracts the virus. SARS-CoV-2, also known as COVID-19, was declared an international emergency disease by the World Health Organization (WHO) only one month after its existence was known [2,3]. Although it was not the first disease to be classified by the WHO as an international emergency disease, its appearance was an unprecedented and global milestone worldwide due to the health, social and economic consequences of the pandemic [4], this has led to a change in certain habits in the child and adolescent population. Some of these modified habits are related to the time dedicated to the practice of daily physical activity as well as the time spent using new technologies [3,5].

According to the latest information provided by the WHO so far, risk factors for severe SARS-CoV-2 disease include age, high blood pressure, heart or lung problems, diabetes, cancer or obesity [6,7]. Some of these risk factors for serious disease are closely linked to sedentary lifestyles and physical inactivity as well as to the dietary habits of the population. Spain has one of the highest prevalences of childhood overweight and obesity in the European Union, being one of the most important risk factors in the paediatric population [8]. In addition to this risk factor, paediatric COVID-19 patients have unusually low vitamin D levels [9], which is an essential vitamin for muscle and bone function in relation to physical activity [10].

This is why the school-age population presents values of 85% in girls and 78% in boys that do not perform the minimum minutes of daily physical activity established by the WHO for their age range [11], a direct consequence of sedentary lifestyles and excessive time spent using technology. If these low levels of physical activity are combined with inadequate eating habits where ultra-processed foods are prevalent, an obesogenic environment is generated in children, which leads to increased rates of overweight and obesity and further accentuates the few minutes a day devoted to physical activity [5,12].

This international institution recommends a minimum of 60 min of moderate to vigorous intensity physical activity (MVPA) per day for school-aged children. It ensures that any physical activity that exceeds these 60 min will be of greater benefit to the child’s health. Similarly, the WHO recommends that children between 5 and 12 years of age should be exposed to screens and new technologies for between 60 and 90 min per day [11,13].

However, these recommendations are not currently being followed by the school population between 9 and 15 years of age and this situation has been accentuated during the periods of confinement in the different countries due to SARS-CoV-2 [14]. This age group has a higher sedentary time and a decrease in physical activity outside the school context, especially in girls and in schoolchildren who already have problems of overweight or obesity compared to schoolchildren with Body Mass Index (BMI) values that are in the normal range [15]. Similarly, exposure times to screens and new technologies are above the WHO recommendations. Both factors constitute the so-called “technological sedentary lifestyle” [5,16] and may be associated with other health problems such as isolation, poor social relations, sleep disturbances, endocrine, musculoskeletal or cardiovascular disorders [12].

There is currently little research addressing SARS-CoV-2 in paediatric patients (0–18 years). The seroprevalence rate in the paediatric population is estimated to be 3.9% [17] in Spain and 1.56% worldwide [18]. However, there are no official data on paediatric COVID-19 seroprevalence and mortality provided by the epidemiological agencies of the countries and regions of the world [19].

On the other hand, there is scientific evidence that regular physical activity at a minimum intensity of effort not only has health benefits but can also prevent infectious diseases such as SARS-CoV-2. Recent research shows that regular physical activity of 30 min a day for at least five days a week can reduce the risk of contracting a virus by 31%, reduce the possibility of death from infectious diseases by 37% and can improve the effectiveness of vaccines by up to 40%, as in the case of the SARS-CoV-2 virus [20].

Better physical fitness has also been shown to improve body composition, blood pressure, better heart rate values as well as better respiratory capacity and maximal oxygen consumption (VO_2_ max) of the subject, which mitigates the risk factors for serious COVID-19 disease [21].

In line with the aforementioned research, this research aims to confirm that home confinement, the closure of schools and sports schools due to the health crisis caused by SARS-CoV-2 have led to a significant decrease in the practice of physical activity, contributing to a worsening of their physical condition, an increase in sedentary lifestyles and an increase in the time spent using new technologies.

Therefore, the main objective of this study was to determine the impact of the large-scale confinement due to SARS-CoV-2 on physical fitness values in the 11–12 year-old population. The relationships between physical fitness values, dietary habits and socio-demographic data (e.g., type of housing, place of residence, time spent in physical activity) were also studied. In this research, the physical fitness values established in the dimensions of musculoskeletal capacity (3 items), motor capacity (1 item) and aerobic capacity (1 item) of the Alpha-Fitness Battery were studied [22]: right hand pressure, left hand pressure, long jump with feet together, 4 × 10 m agility speed test and the Leger test.

## 2. Materials and Methods

### 2.1. Study Design

For the development of this research, a descriptive and longitudinal observational study was carried out [23]. The dependent variables of this research were the musculoskeletal capacity by means of the manual pressure capacity and the long jump with feet together, the motor capacity by means of the 4 × 10 m agility speed test and the aerobic capacity with the 20 m round-trip test (Leger’s Test). Likewise, the estimation of the relative VO_2_ max calculated from the results of the aerobic capacity test of the Leger test was also taken into account as a dependent variable [24]. The values were taken according to the Alpha-Fitness Battery, a validated field test for the assessment of health-related physical fitness in children and adolescents [22]. The use of a validated instrument to assess food consumption, habits and practices in 8–11 year-olds [25] and an ad hoc survey for parents or legal guardians to collect socio-demographic data on the study participants as well as data on the different variables under study were the instruments used to define the independent variables.

### 2.2. Participants

The research consisted of a non-probabilistic sample from a primary school in Cantabria (Spain).

In this case, 50 of the 55 children invited to participate in the study took part in the research. The sample was in the sixth year of Primary Education in a public school. The pupils who did not participate in the research had different reasons or did not present the informed consent of their parents or legal guardians. The sample of 50 schoolchildren aged 11–12 (M = 11.40; SD = 0.50) was divided into 33 (66%) boys and 17 (34%) girls. The residential settings of the sample were distributed in an urban setting (56% of the sample), in a semi-urban or residential setting (38%) and a small part of the sample (6%) in a rural setting.

### 2.3. Tools

The Alpha-Fitness Battery (University of Granada, Granada, Spain), a validated field test for the assessment of health-related physical fitness in children and adolescents [22] was the instrument used to collect the research data. This instrument consists of five dimensions: Dimension 1: Tanner Stage (3 items); Dimension 2: Body Composition (5 items); Dimension 3: Musculoskeletal Capacity (3 items); Dimension 4: Motor Capacity (1 item) and Dimension 5: Aerobic Capacity (1 item). Dimensions 3, 4 and 5 and their reference values have been used in this research [26]: manual pressure capacity, long jump with feet together, 4 × 10 m agility speed test and 20 m round-trip test (Leger test). For the performance of the tests and their measurements, the procedures established by the Alpha-Fitness Battery were followed. A Saehan hand-held digital dynamometer (Saehan Corporation, Masan, Korea) was used to measure grip strength from 0 to 90 kg (200 lb). A MEDID 20 m fibreglass (General de Medición SL, Barcelona, Spain) tape measure with centimetric graduation, JETTING 19 cm diameter floor marker discs (Reehut, Xiamen, China) and a JBL 8 inch 112 dB woofer audio system (Harman International Industries, Los Angeles, CA, USA) were also used for the audio projection of the Leger Test [24]. The results obtained in the Leger test, an indirect, maximal and incremental staircase test, were used for the indirect calculation of the VO_2_ max of the research sample [24].

All data collected on dietary habits has been carried out through a validated instrument to assess dietary consumption, habits and practices in children aged 8–11 [25]. It is made up of 42 items distributed in five sections: frequency of food consumption, cooking skills, eating habits, expenditure on food in the school environment and knowledge.

Finally, for the collection of socio-demographic information, time devoted to physical activity, time devoted to sedentary activities, time spent using new technologies and information on the emotional aspects of the children, an ad hoc survey was designed with 50 items, which was completed by the parents or legal guardians.

### 2.4. Procedure

This research is a consequence of an initial investigation that could not be completed due to the SARS-CoV-2 lockdown in Spain. The aim of the initial unfinished research was to carry out a comparative analysis between two groups of sixth-grade Primary School students with respect to anthropometric parameters, physical condition, psychological and emotional aspects such as anxiety, as well as academic results. However, with the outbreak of SARS-CoV-2 this research planning could not be completed and a new research aim was established: to find out the impact of SARS-CoV-2 lockdown on physical fitness in the 11–12 year-old population.

Once the study was authorised by the educational centres and the Inspection Service, a meeting was called with the parents or legal guardians of the children in the 6th year of primary school who would form part of the research sample. This meeting was to take place at the beginning of the academic year. At this meeting, the objectives and process of the research (data collection, analysis techniques and use of the data collected), the confidentiality of the participants, the voluntary nature of the study and the possibility for their children to leave the study at any time they wished without having to justify their withdrawal from the study were explained. All this information was given to the families in writing together with the informed consent form. Once the informed consents had been given by the families or legal guardians of the participating children, the study began with the first data collection. During the 2019–2020 academic year, two data collections were carried out in physical education classes during the weeks of 14 October 2019 and 2 March 2020, as planned.

However, on 15 March 2020, the home lockdown begins in Spain due to the State of Alarm decreed by the health emergency caused by SARS-CoV-2. During this lockdown, all schools in Spain are closed indefinitely [1]. The third data collection immediately after the end of the home lockdown period and the start of the de-escalation period, on 28 May 2020. In order to guarantee the health measures established by the Spanish government to prevent the spread of SARS-CoV-2, the sample was drawn in groups of 6 children at different times of the day and in an outdoor space.

### 2.5. Statistical Analysis

SPSS statistical software (SPSS v.26, IBM Corporation, New York, NY, USA) was used to perform all the statistical analyses of the study. A descriptive analysis of the main variables under investigation was carried out, as well as normality tests of quantitative variables for the testing of hypotheses. The Kolmogorov-Smirnov statistic (*n* > 50) was used for the normality analyses of the whole sample, while the Shapiro-Wilk statistic (*n* < 50) was used for the normality tests by sex. When the *p*-value of the normality tests was significant (*p* < 0.05), the hypothesis that the variable does not have a normal distribution was accepted.

Depending on the nature of the variables and certain assumptions to be met, different tests of independence have been applied for hypothesis testing. In the case of a normal distribution of the quantitative variable in the different categories of the qualitative variable, parametric tests were performed. If there was no normal distribution in the different categories of the qualitative variable, non-parametric tests were performed. Likewise, the type of test performed depended on whether the categorical or qualitative variable has two or more than two categories.

For parametric tests, when the categorical variable has two categories, the Student’s *t*-test was used, and if it has three or more categories, the comparison of means was carried out through the analysis of variance ANOVA. In the non-parametric tests, when the categorical variable has two categories, the Mann-Whitney U test was used, and if it has three or more groups, the Kruskal Wallis test was used. If the assumption of normality was met, the Student’s *t*-test for paired samples was used to check whether there was a statistically significant difference (*p* < 0.05) between the data obtained pre-lockdown and post-lockdown for the different research variables. If this assumption of normality did not occur in the variables, the non-parametric Wilcoxon rank test was used. For independence between qualitative variables, the chi-square test of independence was used.

Finally, two tests were used depending on whether the variables were normally or non-normally distributed to test the correlation or association between the quantitative variables. If both variables were normally distributed, Pearson’s correlation was used, while if they were not normally distributed, Spearman’s correlation was used.

### 2.6. Ethical Aspects

The ethical and deontological principles established by the American Psychological Association were followed at all stages of the research [27], as well as ethical recommendations for educational research [28,29].

Approval of the research protocol was requested from EDUCA’s Ethics Committee, which was approved under code 82019.

## 3. Results

### 3.1. Physical Fitness Variables

#### 3.1.1. Descriptive and Functional Analysis

To test whether there were statistically significant differences between the pre-lockdown and post- lockdown fitness variables studied (Table 1), the Student’s *t*-test for paired samples was used. This test showed results with statistically significant differences (*p* < 0.05) in the mean value of some fitness variables between the two pre-lockdown measurements and the post-lockdown measurement. The results obtained between the first pre-lockdown data collection and the second pre-lockdown data collection showed statistically significant differences in the variables of agility speed, Leger’s test and VO_2_ max but not in the variables of right hand pressure (*p* = 0.572), left hand pressure (*p* = 0.537) nor in the long jump with feet together (*p* = 0.321).

The results obtained from the pre-lockdown 2 and post- lockdown data collection showed statistically significant results for all fitness variables except for right hand pressure (*p* = 0.241).

Similarly, the paired-samples Student’s *t*-test was used to test whether there were statistically significant differences between the pre- lockdown and post- lockdown fitness variables by sex (Table 1). The test results show that there are also statistically significant differences (*p* < 0.05) in the mean value of the variable between the two pre-lockdown measurements and the post-lockdown measurement. These results show differences between sexes with respect to the mean value of the sample. In the case of the results obtained between the first pre-lockdown data collection and the second pre-lockdown data collection, in boys there are no statistically significant differences in right hand pressure (*p* = 0.949), left hand pressure (*p* = 0.609) or in the long jump (*p* = 0.870), while in the case of girls there are no differences in right hand pressure (*p* = 0.308), left hand pressure (*p* = 0.223) nor in the long jump with feet together (*p* = 0.071).

Regarding the results of the T-student test for paired samples between pre-lockdown 2 and post-lockdown, boys present statistically significant differences in all physical fitness variables except for right (*p* = 0.465) and left (*p* = 0.247) hand pressure, while in girls there are no statistically significant differences only in right hand pressure (*p* = 0.228).

#### 3.1.2. Evolution of Physical Fitness Variables

The evolution shown by the physical condition variables measured prior to lockdown experienced small variations with slight improvements in all the mean values of the sample with the exception of the agility speed variable, which did not show any improvement.

**Figure 1:**
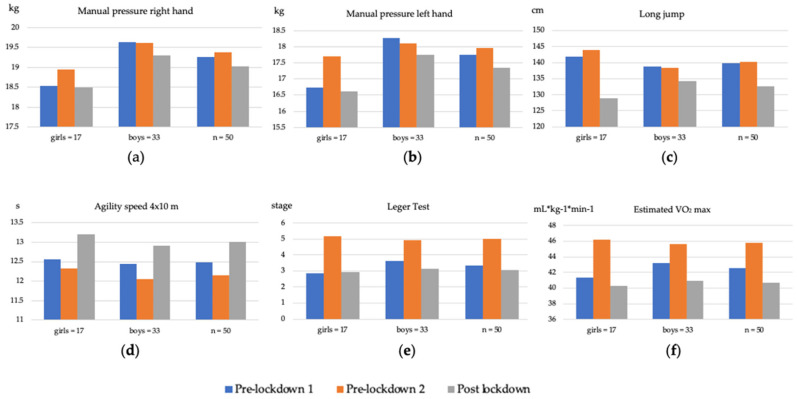
Changes in physical fitness variables in the sample and between genders. (**a**) Changes in the hand pressure variable of the right hand. (**b**) Changes in the hand pressure variable of the left hand. (**c**) Changes in the variable long jump with feet together. (**d**) Evolution of the variable 4 × 10 m agility speed. (**e**) Evolution of the variable of the Leger test. (**f**) Evolution of the relative VO_2_ max variable.

In the case of the results obtained in the physical condition variables between the pre- lockdown data collection and the post-lockdown data collection, they show a worsening in all their mean values as well as by gender, which shows the influence that the period of lockdown due to SARS-CoV-2 has had on the physical condition values of the sample. Table 2 and Figure 1 show the results obtained in pre-lockdown 1, pre-lockdown 2 and post-lockdown for all physical fitness variables. The results are presented for the whole sample as well as broken down by gender. All the results of the fitness variables compared between pre lockdown 2 and post lockdown and shown in Table 2 and Figure 1 have shown significant differences. With the exception of hand pressure of the right hand, which, despite having worsened after lockdown, did not show significant results.

According to the reference values of the Helena study [26], the results of this research show that the mean value of the manual pressure of the pre-lockdown sample was very low in both boys (≤21.4) and girls (≤19.9) and with lower values in the post- lockdown. Regarding the long jump, the mean values were low (136–152) for boys and medium (134–147) for girls and were very low (≤135) for boys and low (119–133) for girls in the post- lockdown. In the agility speed, the boys had average pre-lockdown results (11.8–12.2), while the girls had high results (11.9–12.4). However, the values of this same test post- lockdown have shown low values (12.3–12.9) in boys and a clear decrease in the girls results to very low values (≥21.4). Finally, the results obtained in the aerobic capacity by means of the Leger Test and taking the stages reached as reference values, in the pre- lockdown results the boys obtained average values (5.0–6.0) and the girls very high values (≥5.0). Furthermore, these results in post-lockdown suffered a worsening in the boys to very low values (≤3.0) and a notable decrease in the girls to low values (2.5–2.5).

Finally, the values of VO_2_ max (mL·kg^−1^·min^−1^) have shown good values in the case of boys (45.2–50.9) and higher in the case of girls (≥41.9). Such as the values of the physical condition tests performed, the VO_2_ max also suffered a post- lockdown worsening, obtaining fair values (38.4–45.1) in the case of the boys and a small regression to excellent values in the case of the girls (39.0–41.9).

These results, both pre-lockdown and post-lockdown, show that the sample started the research in poor physical condition and that this situation was aggravated by the period of lockdown as a consequence of SARS-CoV-2.

#### 3.1.3. Relationships between Fitness Variables

Two tests were used to obtain the results of correlation between the physical condition variables of the research between the different points in time at which the data were collected. If the two correlated variables had a normal distribution, the Pearson correlation test was used. For those fitness variables where at least one of them did not have a normal distribution, Spearman’s Rho correlation test was used.

The results of the relationship obtained between the pre-lockdown and post-lockdown physical fitness variables are shown in Table 3.

Between pre-lockdown 2 and post-lockdown there is a very high correlation (0.8 < r < 1) in the results linked to the strength in manual pressure as well as with Leger’s Test and VO_2_ max and a high correlation (0.6 < r < 0.8) with the variables of Leger’s Test and VO_2_ max as well as in the long jump with feet together.

However, it is worth noting the negative or inverse correlation coefficient (−1 < r < 0) that occurs in certain results. This inverse correlation coefficient shows us that the worsening of the results in certain tests carried out in the physical condition variables is associated with the worsening of others. In the case of the correlation result of agility speed, there is a moderate inverse correlation (−0.4 < r < 0.6) with the long jump with feet together, so that the more time spent in the execution of the agility speed test results in a shorter jump distance in the long jump test with feet together (r = −0.543), as well as less distance covered and fewer levels executed in the Leger Test (r = −0.594). These inverse correlations are also reflected in the VO_2_ max which has a decrease in its values after lockdown (r = −0.524). Finally, there is also a moderate inverse correlation (−0.4 < r < 0.6) between VO_2_ max and agility speed (r = −0.507). The results of these correlation tests confirm the influence of some fitness variables on others and the deterioration of their values after the lockdown period.

The rest of the correlation test results on the physical fitness variables of pre-lockdown 2 and post-lockdown have been low (0.2 < r < 0.4) or very low (0 < r < 0.2).

### 3.2. Dietary Habits

#### 3.2.1. Descriptive Analysis

In order to obtain the results related to dietary habits, 12 of the 42 items of the validated instrument for assessing food consumption, habits and practices in children aged 8–11 were taken into account. The 12 items are those directly related to consumption habits: daily glasses of water, daily vegetable dishes or salads, daily pieces of fruit, daily bread, weekly amount of food rich in saturated fat, daily dairy products, weekly fish, daily glasses of soft drinks, weekly legumes consumed, weekly sweets and jelly beans consumed, weekly salty snacks and weekly cakes and pastries and sweet pastries consumed. Two categorical items on the number of meals eaten per day and the type of food consumed in the mid-morning were also taken into account.

These items were analysed before lockdown and during lockdown and a descriptive and comparative analysis of the results obtained was carried out. All the variables evaluated showed higher results between the type of food consumed and its frequency in pre-confinement and during lockdown, except for dairy products consumed daily pre-lockdown (M = 2.48; SD = 0.93) with respect to the lockdown period (M = 2.20; SD = 0.92) and fish consumed weekly pre-lockdown (M = 1.70; SD = 0.90) with respect to that consumed during lockdown (M = 1.60; SD = 0.94).

To obtain the results in the categorical items, cross tables were made between the frequency of food consumed pre-lockdown versus those consumed during lockdown, as well as the number of daily meals eaten. The cross-tabulations showed results with an increase in the number of daily meals eaten during lockdown compared to the pre-lockdown period. There were also changes in certain foods between the pre-lockdown period and during lockdown, an increase in the consumption of salty snacks (5.3%) and bread with additives (10%) and a decrease in the frequency of consumption of foods such as fruit consumed daily (−5.6%) during the lockdown period.

The two categorical items obtained a significant result (*p* < 0.05) according to Pearson’s chi-squared test of independence, so it can be affirmed with 95% confidence that the hypothesis of dependence between the variables analysed in the first categorical item (*p* < 0.030) as well as in the second analysed (*p* < 0.013) is accepted.

#### 3.2.2. Significance Analysis

The Kolmogorov–Smirnov test was used to test the normality of the variables. The test showed significant results (*p* < 0.05) between the pre-lockdown and post-lockdown variables, thus accepting the hypothesis that the variable does not have a normal distribution. With this result of non-normality of the variables, the Wilcoxon non-parametric test of related samples was carried out, which yielded results that indicate the existence of statistically significant differences (*p* < 0.05) between three variables pre-lockdown with respect to post-lockdown in the mean value of the variable. These three variables are daily fruit (*p* < 0.048), daily dairy consumed (*p* < 0.038) and weekly salty snacks consumed (*p* < 0.021).

#### 3.2.3. Correlation Results

The post-lockdown physical condition variables showed low (0.2 < r < 0.4) and very low (0 < r < 0.2) correlations with the pre-lockdown dietary variables. In these results, negative or inverse correlation results also appear with low (−0.4 < r < −0.2) and very low (−0.2 < r < 0) results, so we can affirm that there has been no significant relationship or influence between these variables in this research.

The same low and very low correlation results were found between the post-lockdown physical condition variables and the dietary variables during lockdown. Only the significant correlations between right hand pressure (r = 0.402) and left hand pressure (r = 0.450) with respect to the food variable referring to the consumption of vegetable dishes or salads consumed, in which an increase in the consumption of these foods is associated with lower hand pressure values, should be highlighted. Similar results also appear for the agility speed test variable in relation to the number of glasses of soft drinks consumed per day (r = 0.404). Table 4 shows the details of the correlation results obtained.

### 3.3. Family Habits

#### 3.3.1. Descriptive Analysis

In order to obtain the data related to family habits, a family survey made up of categorical variables was used. A basic descriptive analysis was carried out on these variables on the time spent by the sample on physical activity, the time spent on sedentary activities such as exposure to screens and the analysis of the time spent on other types of sedentary activities during the period of lockdown.

The data obtained on the time spent by the sample on screen exposure during the period of lockdown show that 52% used the video console for more than 60 min a day, 50% the television, 48% the computer, 30% the tablet and 26% the mobile phone.

The time related to educational and cultural activities indicates that 82% of the sample spent more than 60 min doing homework and 30% more than 146 min, 38% spent between 16 and 30 min a day reading, 2% played musical instruments and 8% did artistic activities for more than 60 min.

The time the sample spent resting was also recorded in terms of hours of sleep. The results showed that 20% of the sample slept 8 h a day, 40% slept for 9 h and 38% slept for 10 h a day.

With regard to the time spent by the sample on physical activity, it was observed that in any range of frequency of physical activity, there was a decrease in the time spent. Four percent of the sample did not engage in any type of physical activity before lockdown, a percentage that has increased to 32% of the sample. Similarly, the frequency of children who were physically active 2 to 3 times a week decreased by 10% during the period of lockdown, 14% among children who were physically active 4 to 5 days a week and children who were physically active 6 to 7 days a week decreased from 10% pre-lockdown to 6% during lockdown.

The place of residence where the children in the sample lived during lockdown was also analysed. The environment and the space available for physical activity was considered an important element for the research and some of the results of the research were conditioned by these variables. While 6% of the sample resided in a rural environment during lockdown, 38% resided in a semi-urban or residential environment and 56% of the sample resided in an urban environment.

There were also differences according to the size of the housing in which the children resided during lockdown. In this case, 36% of the sample resided in a house with a garden during lockdown, 28% resided in a flat between 91 and 120 m^2^, 28% in a flat between 61 and 90 m^2^ and 8% of the sample resided in a flat of less than 60 m^2^.

The participants who showed better fitness values were those who lived in an urban environment compared to those who lived in a semi-urban or residential environment or in a rural environment. They performed better on all fitness variables assessed except for hand pressure in both the left and right hand.

The results do not show that the size of the housing in which the children resided during the period of lockdown had a significantly influenced, which shows that the decrease in minutes of physical activity performed during confinement was not conditioned by the space available to the children in their homes for physical activity.

#### 3.3.2. Statistically Significant Differences

The Mann-Whitney U test and the Kruskal-Wallis H test were used to test for significant differences between the physical fitness variables and the sociodemographic variables collected by the family survey. These tests of independence have not shown that there are statistically significant differences (*p* > 0.05) between the post-lockdown physical fitness variables and the socio-demographic variables. The results show three exceptions such as right hand pressure (*p* = 0.039), left hand pressure (*p* = 0.02) and long jump (*p* = 0.045) with the type of dwelling in which the children resided during the period of lockdown.

#### 3.3.3. Post-Lockdown Results

An analysis was made of the results obtained for the post-lockdown physical fitness variables in relation to the family habit variables analysed. This analysis focused on the variables of the family survey related to the child’s physical activity and other sedentary activities during lockdown.

The most significant results of the physical condition variables during lockdown in relation to the frequency of physical activity (Table 5), is the improvement of some of their values in the children who did not practice physical activity prior to lockdown, which shows that during the period of lockdown they increased their physical activity time.

A descriptive and comparative analysis of the results obtained for the different physical condition variables analysed during lockdown are also shown in relation to the time spent by the children in the sample on activities related to the use of new technologies during lockdown. The results show that the children who spent the most time using new technologies were the ones who showed the worst physical fitness values in some variables. In this type of sedentary activities, we highlight the use of tablets or computers, video game consoles, television and mobile phones. The results show that children who have used technological devices have had worse results in some of the physical fitness variables analysed. This worsening of results presents different values depending on the technological device used. There was a decrease in the values of kg mobilised in hand pressure, both right and left, in the sample that used video game consoles (−0.98 and −0.44, respectively), computers (−0.21 in right hand) and tablets (−2.70 and −0.53, respectively) in particular. The results for the long jump with feet together only showed a worsening for the children who used the television (−2.98) to a greater extent. However, agility speed (+0.86), Leger’s test (−1.55) and VO_2_ max (−4.4) showed worse values in all members of the sample who used technological devices during the lockdown period.

Similarly, an analysis of the results obtained for the different physical condition variables during lockdown has been carried out in relation to the time that the children participating in the research have dedicated to other sedentary activities during the period of lockdown. These sedentary activities refer to homework, reading, playing musical instruments or artistic activities. As with the time children spent on the use of new technologies, the physical condition variables showed worse values for the children in the sample who spent more minutes per day on this type of activity.

## 4. Discussion

The aim of this study was to determine the impact that confinement due to SARS-CoV-2 had on physical fitness parameters in children aged 11–12.

Through this research it has been shown that there have been modifications in the results of the physical condition values between pre-lockdown and post-lockdown. These results show significant differences both in the total sample and in both sexes. There were significant decreases in the number of kg that were able to mobilise with both the right and left hand. There has also been a significant decrease in the distance of the long jump test with feet together, a decrease in the stages reached in the Leger test as well as a pronounced decrease in their VO_2_ max values. On the other hand, and also with worse results between pre-lockdown and post-lockdown, the time dedicated to the execution of the agility test has increased. These significant results are evidence of a notable loss of physical condition of the sample after the period of high lockdown in Spain.

The results show that all the fitness variables assessed showed improvements in their values, and therefore also in their performance, between the first two data collections prior to the lockdown. However, some of these improvements were not significant. These non-significant results were the kg of pressure in both hands, which obtained slightly better values between pre-lockdown 1 and pre-lockdown 2 by increasing the kg mobilised in the test. These non-significant improvements occurred in the total values of the sample as well as in both sexes. There was also a slight non-significant improvement in the distance of the long jump with feet together in the total sample and in the boys, increasing the jump distance. However, there was a significant improvement in the case of the girls (*p* = 0.0039) with an improvement of 2 cm between pre-lockdown 1 and pre-lockdown 2. The rest of the physical condition variables did show significant improvements. The time taken to perform the agility speed test decreased significantly, except in the girls’ results (*p* = 0.0071), which reduced the time taken to perform the test slightly. The stages of the Leger test were significantly higher for the total sample as well as in both sexes. These improvements in the fitness values of the sample also translated into significant increases in the VO_2_ max values of the sample and of both sexes.

However, these results suffered a significant reversal in their values after the great Spanish lockdown. Worse results were obtained in all the tests performed with respect to pre-lockdown 2. The results show significant worsening in all the results of the physical condition variables except for the manual pressure of the right hand. However, although not significantly, the right hand pressure values were also lower than the previous data collection, mobilising less kg in the test. There was a significant decrease in the kg of pressure mobilised in the manual test of the left hand, a decrease in the distance of the long jump at fair feet as well as a reduction in the stages reached in the Leger Test. Likewise, significantly worse results were obtained in the agility speed test, producing an increase in the execution time of the test. All these post lockdown results had as a consequence a worsening in the VO_2_ max values, being lower in the total sample as well as by sexes with respect to the previous data collection.

This study has also shown that the sample has modified its habits during the period of lockdown compared to its pre-lockdown habits. During lockdown, there was an increase in the time spent using new technologies, an increase in the time spent on sedentary activities and a notable decrease in the time spent daily on physical activity and in the number of days per week spent doing physical activity. Similarly, there was evidence of a change in dietary habits during the period of lockdown, with an increase in the intake of foods rich in saturated fats, sugars and snacks and a decrease in the intake of fruit and legumes. The results show worse physical fitness values in children who lived in small dwellings without a garden during lockdown and in children whose parents were less educated or who did not engage in regular physical activity prior to the SARS-CoV-2 pandemic.

Different studies and institutions confirm that children and adolescents do not comply with the recommendations for time dedicated to physical activity and the eradication of sedentary behaviour [30]. This aspect together with the changes in habits brought about by the SARS-CoV-2 health crisis [5,31,32] can lead to a decrease in children’s physical condition as well as changes in their body parameters. Similarly, this study shows that the time devoted to physical activity during lockdown falls short of the minimum time recommended by the WHO [33,34], this has led to a lower level of physical fitness and a worsening of test values.

In this context, numerous studies and research suggest that regular physical activity and a good level of physical fitness is instrumental in preventing COVID-19 infection as well as reducing the risks of the disease. Regular physical activity leads to improved anti-inflammatory, anti-fibrotic and antioxidant processes that can mitigate the negative effects that COVID-19 can have on the body [20,35,36].

In addition, some studies confirm that having a good level of physical fitness can be associated with high VO_2_ max values, a basic indicator of a person’s health, which can be affected by COVID-19 with a decrease of up to 10% of pre-infection values [37]. In the opposite case, low VO_2_ max levels are associated with low levels of physical fitness, high prevalence of obesity and lower immune systems than the regularly active population [38].

This research also confirms that a high percentage of children participating in this study exceed the WHO recommended minutes of consumption of new technologies [11], this has been to the detriment of the practice of physical activity and, as a consequence, a lower level of physical fitness. Some countries, such as China, have already begun to limit the use of technologies, such as online video games, to prevent addiction and sedentary lifestyles among minors [39]. This situation, together with the time spent in other sedentary activities, has become an important determinant of low levels of physical fitness among children, as well as the existence of changes in body parameters, thus favouring the prevalence of childhood overweight and obesity [39,40]. Another important factor for maintaining a good level of physical fitness and favouring the body’s recovery processes is rest. The WHO recommends that children of this age should sleep at least 11 h a day, a situation that was not met by any member of the sample in this study.

In addition to the changes evidenced in the practice of physical activity in this research, changes in habits produced by the lockdown due to SARS-CoV-2 have also been shown [5,31,32,41] in terms of food. There has been an increased number of meals per day and an increased intake of foods rich in saturated fats and sugars, thus favouring the change in children’s body parameters [42,43] and as a consequence of the possible loss of physical fitness.

Finally, it is worth highlighting the limitations of this research. The first limitation of this research is the sample size. Due to the outbreak of SARS-CoV-2 and the modification of the original research, the sample size was reduced. For this reason, the results obtained in this research cannot be generalised to the entire population aged 6–12 years. The second limitation, also related to the sample, is the difference in sample size by gender.

However, it should be taken into account that the profile of the sample participants is very similar to the samples cited in the different studies mentioned above as well as in other governmental or institutional documents. Among these documents, the results of the latest survey by the Spanish National Statistics Institute (INE) in 2020 [43] should be highlighted. This survey is used for the elaboration of the EUROSTAT reports [44]. Both documents also show a parallelism in the characteristics of the sample of this research with the general population of the same age group in relation to the values of physical activity and eating habits. For this reason, and although this limitation means that it is not possible to generalise the results and evidence found, the study provides unique evidence and information. The evidence found goes deeper into results obtained by direct methods that are unlikely to be repeated in similar circumstances. Moreover, this study can become an excellent indicator of the consequences that such measures of social isolation, whether as a consequence of lockdown or any other circumstance, can have on the population of this age group.

Without being able to generalise the results of this research to the general population of this age group, this research demonstrates the need to continue strengthening and promoting effective strategies to encourage the practice of healthy and regular physical activity. Only by working on effective strategies can children’s adherence to regular physical activity be achieved. This adherence would mean that in the event of a new home lockdown such as the one described in this research, it would not lead to an abandonment of physical activity or a worsening of variables related to their physical condition and health. The adherence of children and adolescents to physical activity would mean that in the event of a new home lockdown, they would seek new ways to continue practising physical activity.

These strategies should also encourage the acquisition and maintenance of healthy eating habits and the responsible use of technology, especially in the aftermath of situations such as the major SARS-CoV-2 lockdown. These strategies should be implemented by the main agents of the child’s socialisation. In the first place, and as the most important agent of socialisation for children, the family. The family model becomes a point of reference for children at a very early age, which is why the family model of responsible consumption of new technologies, correct eating habits and regular physical activity will be the most effective strategy for avoiding a sedentary lifestyle and unhealthy eating habits.

The second socialising agent that can contribute to improving the values of physical fitness and healthy habits in children is the school. Educational centres in general, and the physical education area in particular, should encourage the regular practice of physical activity with the aim of improving health. Some strategies that can help to improve levels of physical fitness and healthy habits are healthy lunches, active breaks or awareness-raising days about overweight, obesity and overweight children. Similarly, a good range of extracurricular school sports activities will reinforce all the actions and strategies worked on at school.

And finally, sports organisations. These entities, in close relationship with families and schools, should become a reinforcement of healthy behaviours and habits in terms of physical activity, nutrition and occupation of leisure time for children and adolescents.

There is an urgency for the design and implementation of a strategic plan due to the negative consequences that lockdown has had on the sample in terms of time spent in physical activity and their low levels of physical fitness and the risks that this entails.

## 5. Conclusions

This research shows results that demonstrate a worsening of the physical condition of 11 and 12 year old Spanish boys and girls as a consequence of the impact of the SARS-CoV-2 closure. The loss of physical fitness level is observed in values that have worsened with a decrease in their aerobic capacity (−39.24%), a decrease in their upper body maximum strength values (−1.89% in the right hand and −3.51% in the left hand), a decrease in their lower body explosive strength values (−5.69%) and an increase in their agility speed times (6.68%) between pre-lockdown and post-lockdown. This worsening is also shown in the values of one of the most important health indicators such as VO_2_ max with a decrease in its values of 12.61%. The importance of a good physical condition to minimise the impact that a contagious disease can have on people as well as their prevention of infection, especially a disease such as COVID-19, should be emphasised.

This deterioration in their level of physical fitness has had a multifactorial origin consisting of an excess of time spent using new technologies, changes in their eating habits, as well as a decrease in the time spent in physical activity during lockdown. It should be noted that the children in the sample already had low levels of physical fitness, so the period of lockdown due to SARS-CoV-2 has only aggravated an already worrying situation. In relation to gender, there is a more pronounced drop in the level of physical fitness and in the values of certain tests in girls than in boys, but even so, they continue to have better levels of physical fitness than boys. However, it should be highlighted that one of the limitations of the research is the difference in sample size by gender.

## Figures and Tables

**Table 1:** Significance results for fitness variables as a function of data collection and gender. Student’s *t*-test for paired samples.

Variables	Pre Lockdown 1–Pre Lockdown 2			Pre Lockdown 1–Post Lockdown			Pre Lockdown 2–Post Lockdown		
	*n*	Boys	Girls	*n*	Boys	Girls	*n*	Boys	Girls
RHMP	0.572	0.949	0.308	0.486	0.460	0.933	0.241	0.465	0.228
LHMP	0.537	0.609	0.223	0.201	0.161	0.806	**0.018**	0.247	**0.023**
LJ	0.321	0.870	0.039	**0.004**	0.090	**0.016**	**0.000**	**0.041**	**0.002**
AS	**0.000**	**0.001**	0.071	**0.002**	**0.003**	0.173	**0.000**	**0.000**	**0.016**
LT	**0.000**	**0.000**	**0.000**	0.130	0.032	0.718	**0.000**	**0.000**	**0.000**
VO_2_M	**0.000**	**0.002**	**0.000**	**0.000**	**0.000**	0.207	**0.000**	**0.000**	**0.000**

Note. n = 50; boys = 33; girls = 17. RHMP: right hand manual pressure; LHMP: left hand manual pressure; LJ: long jump; AS: agility speed 4 × 10; LT: Leger’s test; VO_2_M: relative maximal oxygen uptake. The results shown in bold show statistically significant differences.

**Table 2:** Comparison of the results of the physical condition variables between pre-lockdown 1, pre-lockdown 2 and post-lockdown. Descriptive analysis.

Variables	Pre Lockdown 1			Pre Lockdown 2			Post Lockdown		
	*n*	Boys	Girls	*n*	Boys	Girls	*n*	Boys	Girls
RHMP	19.25 ± 3.30	19.63 ± 3.01	18.52 ± 3.79	19.38 ± 3.62	19.61 ± 3.30	18.94 ± 4.26	19.02 ± 4.18	19.29 ± 4.10	18.48 ± 4.40
LHMP	17.74 ± 3.32	18.26 ± 3.11	16.74 ± 3.60	17.96 ± 3.56	18.09 ± 3.27	17.70 ± 4.14	17.35 ± 3.76	17.74 ± 3.86	16.60 ± 3.54
LJ	139.75 ± 18.38	138.68 ± 17.07	141.87 ± 21.19	140.22 ± 20.16	138.33 ± 19.49	143.88 ± 21.54	132.67 ± 18.28	134.34 ± 17.79	128.85 ± 19.49
AS	12.48 ± 0.84	12.44 ± 0.78	12.56 ± 0.98	12.14 ± 0.90	12.04 ± 0.86	12.32 ± 0.97	13.01 ± 1.43	12.91 ± 1.03	13.19 ± 2.03
LT	3.35 ± 1.97	3.60 ± 2.13	2.84 ± 1.52	5.02 ± 2.50	4.93 ± 2.52	5.17 ± 2.51	3.05 ± 1.51	3.12 ± 1.44	2.91 ± 1.66
VO_2_M	42.56 ± 4.98	43.16 ± 5.49	41.33 ± 3.55	45.81 ± 6.47	45.60 ± 6.53	46.22 ± 6.52	40.68 ± 4.09	40.90 ± 4.16	40.27 ± 4.03

Note. Data is presented as mean ± standard deviation. n = 50; boys = 33; girls = 17. RHMP: right hand manual pressure; LHMP: left hand manual pressure; LJ: long jump; AS: agility speed 4 × 10; LT: Leger’s test; VO_2_M: relative maximal oxygen uptake.

**Table 3:** Correlation results of pre-lockdown and post-lockdown physical fitness variables. Pearson’s Correlation and Spearman’s Rho tests.

Pre Lockdown 2	Post Lockdown					
	RHMP	LHMP	LJ	AS	LT	VO_2_M
RHMP	**0.852**	**0.825**	−0.013	0.028	0.036 ^1^	0.004 ^1^
LHMP	**0.849**	**0.888**	−0.129	−0.008	−0.025 ^1^	−0.054 ^1^
LJ	−0.129	−0.052	**0.709**	**−0.664**	**0.601 ^1^**	0.520 ^1^
AS	0.105	0.022	−0.543	**0.683**	−0.594 ^1^	−0.524 ^1^
LT	−0.118	−0.053	0.421	−0.536	**0.794 ^1^**	**0.808 ^1^**
VO_2_M	−0.145	−0.089	0.400	−0.507	**0.781 ^1^**	**0.809 ^1^**

Note. Data is presented as correlation coefficient. RHMP: right hand manual pressure; LHMP: left hand manual pressure; LJ: long jump; AS: agility speed 4 × 10; LT: Leger’s test; VO_2_M: relative maximal oxygen uptake. ^1^ Results obtained with Spearman’s Rho test. The results shown in bold are moderately correlated.

**Table 4:** Correlation results of post lockdown physical fitness variables with pre- and during lockdown feeding variables. Pearson’s Correlation Tests.

Post Lockdown	W	VS	Pre Lockdown 2									
			FR	B	FHSF	MP	FH	R	L	SC	SS	CSD
RHMP	−0.168	0.122	0.244	−0.076	−0.077	−0.158	−0.245	−0.029	−0.074	0.122	−0.007	0.219
LHMP	0.047	0.103	0.312	−0.036	−0.015	−0.098	−0.206	−0.038	−0.027	0.136	−0.059	0.104
LJ	0.088	0.050	0.116	−0.011	0.096	0.080	0.199	−0.030	0.079	0.026	−0.205	0.049
AS	0.163	−0.056	−0.179	−0.029	0.068	−0.034	−0.054	−0.073	0.038	−0.114	0.188	−0.094
LT	0.187	0.171	0.209	−0.123	−0.032	0.149	0.063	−0.033	0.156	−0.019	−0.227	0.087
VO_2_M	0.228	0.151	0.206	−0.121	−0.097	0.053	0.051	−0.069	0.066	−0.053	−0.268	0.102
**Post Lockdown**	**W**	**VS**	**During Lockdown**									
			**FR**	**B**	**FHSF**	**MP**	**FH**	**R**	**L**	**SC**	**SS**	**CSD**
RHMP	0.006	**0.402**	0.294	−0.179	−0.095	−0.196	−0.038	0.274	0.030	0.079	0.110	0.270
LHMP	0.193	**0.450**	0.349	−0.171	−0.001	−0.013	−0.036	0.247	0.022	0.210	0.101	0.331
LJ	0.137	0.035	0.053	0.005	−0.015	0.048	0.166	−0.304	−0.185	−0.025	−0.142	−0.009
AS	0.180	0.098	−0.122	−0093	−0145	−0.161	−0.047	**0.404**	0.045	−0.114	0.120	0.068
LT	0.233	0.132	0.144	0.036	0.015	0.226	0.271	−0.311	0.277	0.080	−0.095	0.044
VO_2_M	0.220	0.043	0.132	0.049	−0.089	0.188	0.236	−0.367	0.202	0.028	−0.227	0.005

Note. Data is presented as correlation coefficient. RHMP: right hand manual pressure; LHMP: left hand manual pressure; LJ: long jump; AS: agility speed 4 × 10; LT: Leger’s test; VO_2_M: relative maximal oxygen uptake. W: glasses of water/day; VS: vegetable dishes or salads/day; FR: fruits/day; B: bread/day; FHSF: food high in saturated fat/week; MP: dairy/day; FH: fish/week; R: glasses of soft drink/day; L: pulses/week; SC: sweets/week; SS: salty snacks/week; CSD: cakes and sweet pastries/week. The results shown in bold are moderately correlated.

**Table 5:** Results of pre and post-lockdown physical fitness variables in relation to the frequency of children who were not physically active. Descriptive analysis.

PA	RHMP	LHMP	LJ	AS	LT	VO_2_M
Pre lockdown	14.50 ± 0.00	14.75 ± 3.04	132.0 ± 0.00	13.44 ± 0.127	1.5 ± 0.707	38.40 ± 1.69
Durante lockdown	17.68 ± 4.61	16.06 ± 3.45	128.85 ± 12.97	13.50 ± 1.787	2.7 ± 1.449	40.57 ± 3.69

Note. Data is presented as mean ± standard deviation. PA: physical activity; RHMP: right hand manual pressure; LHMP: left hand manual pressure; LJ: long jump; AS: agility speed 4 × 10; LT: Leger’s test; VO_2_M: relative maximal oxygen uptake.

## Data Availability

Data sharing not applicable.
